# 1-[(Cyclo­propyl­meth­oxy)meth­yl]-6-(3,4-dimeth­oxy­benzyl)-5-ethyl-1,2,3,4-tetra­hydro­pyrimidine-2,4-dione ethanol hemisolvate

**DOI:** 10.1107/S1600536811055693

**Published:** 2012-01-11

**Authors:** Nasser R. El-Brollosy, Ali A. El-Emam, Omar A. Al-Deeb, Seik Weng Ng

**Affiliations:** a Department of Pharmaceutical Chemistry, College of Pharmacy, King Saud University, Riyadh 11451, Saudi Arabia; b Department of Chemistry, University of Malaya, 50603 Kuala Lumpur, Malaysia; c Chemistry Department, Faculty of Science, King Abdulaziz University, PO Box 80203 Jeddah, Saudi Arabia

## Abstract

The asymmetric unit of the compound, C_20_H_26_N_2_O_5_·0.5C_2_H_5_OH, consists of two tetra­hydro­pyrimidine-2,4-dione mol­ecules and an ethanol mol­ecule. The pyrimidine rings are nearly planar (r.m.s. deviation = 0.006 Å in one mol­ecule and 0.009 Å in the other); the C atom at the 5-position deviates by 0.083 (3) Å [0.064 (3) Å in the second mol­ecule] from the mean plane and the C atom at the 6-position by 0.034 (3) Å [0.082 (3) Å in the second mol­ecule]. In each mol­ecule, the benzene ring is nearly perpendicular to the pyrimidine ring, the dihedral angle is 88.51 (8)° in one mol­ecule and 84.70 (8)° in the other. The amino group of each tetra­hydro­pyrimidine-2,4-dione mol­ecule is a hydrogen-bond donor to the exocyclic O atom at the 2-position of an adjacent mol­ecule, the hydrogen bond generating an inversion dimer in each case. The ethanol mol­ecule forms a hydrogen bond to the meth­oxy O atom of one of two independent mol­ecules.

## Related literature

For the synthesis, see: El-Brollosy *et al.* (2008 ▶).
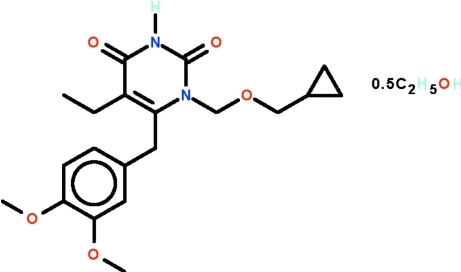


## Experimental

### 

#### Crystal data

C_20_H_26_N_2_O_5_·0.5C_2_H_6_O
*M* *_r_* = 397.46
Monoclinic, 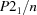

*a* = 14.0251 (5) Å
*b* = 9.4285 (3) Å
*c* = 30.8606 (12) Å
β = 91.580 (3)°
*V* = 4079.3 (3) Å^3^
*Z* = 8
Mo *K*α radiation
μ = 0.09 mm^−1^
*T* = 100 K
0.30 × 0.20 × 0.05 mm

#### Data collection

Agilent SuperNova Dual diffractometer with an Atlas detector
Absorption correction: multi-scan (*CrysAlis PRO*; Agilent, 2010 ▶) *T*_min_ = 0.972, *T*_max_ = 0.995
41334 measured reflections
9419 independent reflections
6406 reflections with *I* > 2σ(*I*)
*R*_int_ = 0.052

#### Refinement

*R*[*F* ^2^ > 2σ(*F* ^2^)] = 0.056
*wR*(*F* ^2^) = 0.166
*S* = 1.03
9419 reflections
526 parameters
3 restraints
H atoms treated by a mixture of independent and constrained refinement
Δρ_max_ = 0.61 e Å^−3^
Δρ_min_ = −0.31 e Å^−3^

### 

Data collection: *CrysAlis PRO* (Agilent, 2010 ▶); cell refinement: *CrysAlis PRO*; data reduction: *CrysAlis PRO*; program(s) used to solve structure: *SHELXS97* (Sheldrick, 2008 ▶); program(s) used to refine structure: *SHELXL97* (Sheldrick, 2008 ▶); molecular graphics: *X-SEED* (Barbour, 2001 ▶); software used to prepare material for publication: *publCIF* (Westrip, 2010 ▶).

## Supplementary Material

Crystal structure: contains datablock(s) global, I. DOI: 10.1107/S1600536811055693/xu5424sup1.cif

Structure factors: contains datablock(s) I. DOI: 10.1107/S1600536811055693/xu5424Isup2.hkl

Supplementary material file. DOI: 10.1107/S1600536811055693/xu5424Isup3.cml

Additional supplementary materials:  crystallographic information; 3D view; checkCIF report

## Figures and Tables

**Table 1:** Hydrogen-bond geometry (Å, °)

*D*—H⋯*A*	*D*—H	H⋯*A*	*D*⋯*A*	*D*—H⋯*A*
N1—H1⋯O1^i^	0.88 (1)	1.96 (1)	2.839 (2)	177 (2)
N3—H3⋯O6^ii^	0.88 (1)	1.92 (1)	2.799 (2)	175 (2)
O11—H11⋯O10	0.85 (1)	2.08 (1)	2.927 (2)	178 (1)

Symmetry codes: (i) 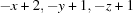; (ii) 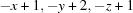.

**Fig. 1.**
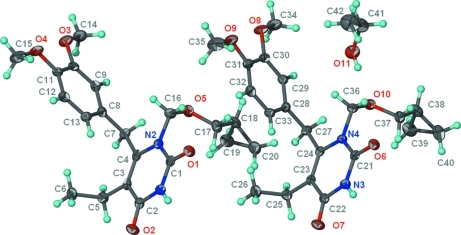
Thermal ellipsoid plot (Barbour, 2001) of C20H26N2O5.0.5C2H5OH at the 70% probability level; hydrogen atoms are drawn as spheres of arbitrary radius.

**Fig. 2.**
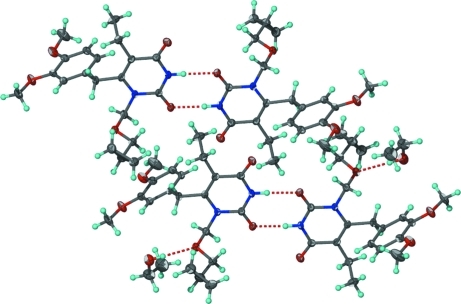
Dimeric hydrogen-bonded structure.

## supplementary crystallographic information

### Comment

The compound was synthesized for an evaluation of its anti-viral activity against HIV-1 (El-Brollosy, 2008). The asymmetric unit of C_20_H_26_N_2_O_5_^.^0.5(C_2_H_5_OH) (Scheme I) consists of two tetrahydropyrimidine-2,4-dione molecules and an ethanol molecule. The pyrimidine rings are planar; the C atom at the 5-position deviates by 0.083 (3) Å (0.064 Å in the second molecule) from the mean plane and the C atom at the 6-position by 0.034 (3) Å (0.082 (3) Å in the second molecule) (Fig. 1). The amino group is hydrogen-bond donor to the exocyclic O atom at the 2-position, the hydrogen bond generating a centrosymmetric dimer. The ethanol molecule forms a hydrogen bond to the methoxy O atom of one of two independent molecules (Table 1, Fig. 2).

### Experimental

The compound was synthesized by using a reported method (El-Brollosy, 2008), and was recrystallized from ethanol.

### Refinement

Carbon-bound H-atoms were placed in calculated positions [C–H 0.95 to 0.98 Å, *U*_iso_(H) 1.2 to 1.5*U*_eq_(C)] and were included in the refinement in the riding model approximation.

The amino and hydroxy H-atoms were located in a difference Fourier map, and were refined with distance restraints of N–H 0.88±0.01 and O–H 0.84±0.1 Å; their temperature factors were refined.

### Crystal data

**Table:**

C_20_H_26_N_2_O_5_·0.5C_2_H_6_O	*F*(000) = 1704
*M**_r_* = 397.46	*D*_x_ = 1.294 Mg m^−^^3^
Monoclinic, *P*2_1_/*n*	Mo *K*α radiation, λ = 0.71073 Å
Hall symbol: -P 2yn	Cell parameters from 8025 reflections
*a* = 14.0251 (5) Å	θ = 2.4–27.5°
*b* = 9.4285 (3) Å	µ = 0.09 mm^−^^1^
*c* = 30.8606 (12) Å	*T* = 100 K
β = 91.580 (3)°	Prism, colorless
*V* = 4079.3 (3) Å^3^	0.30 × 0.20 × 0.05 mm
*Z* = 8	

### Data collection

**Table:**

Agilent SuperNova Dual diffractometer with an Atlas detector	9419 independent reflections
Radiation source: SuperNova (Mo) X-ray Source	6406 reflections with *I* > 2σ(*I*)
Mirror	*R*_int_ = 0.052
Detector resolution: 10.4041 pixels mm^-1^	θ_max_ = 27.6°, θ_min_ = 2.4°
ω scan	*h* = −18→18
Absorption correction: multi-scan (CrysAlis PRO; Agilent, 2010)	*k* = −12→12
*T*_min_ = 0.972, *T*_max_ = 0.995	*l* = −39→40
41334 measured reflections	

### Refinement

**Table:**

Refinement on *F*^2^	Primary atom site location: structure-invariant direct methods
Least-squares matrix: full	Secondary atom site location: difference Fourier map
*R*[*F*^2^ > 2σ(*F*^2^)] = 0.056	Hydrogen site location: inferred from neighbouring sites
*wR*(*F*^2^) = 0.166	H atoms treated by a mixture of independent and constrained refinement
*S* = 1.02	*w* = 1/[σ^2^(*F*_o_^2^) + (0.074*P*)^2^ + 1.8464*P*] where *P* = (*F*_o_^2^ + 2*F*_c_^2^)/3
9419 reflections	(Δ/σ)_max_ = 0.001
526 parameters	Δρ_max_ = 0.61 e Å^−^^3^
3 restraints	Δρ_min_ = −0.31 e Å^−^^3^

### Special details

**Table:**

Geometry. All e.s.d.'s (except the e.s.d. in the dihedral angle between two l.s. planes) are estimated using the full covariance matrix. The cell e.s.d.'s are taken into account individually in the estimation of e.s.d.'s in distances, angles and torsion angles; correlations between e.s.d.'s in cell parameters are only used when they are defined by crystal symmetry. An approximate (isotropic) treatment of cell e.s.d.'s is used for estimating e.s.d.'s involving l.s. planes.

### Fractional atomic coordinates and isotropic or equivalent isotropic displacement parameters (Å^2^)

**Table:**

	*x*	*y*	*z*	*U*_iso_*/*U*_eq_	
O1	0.98988 (10)	0.46926 (14)	0.55571 (4)	0.0200 (3)	
O2	1.09507 (10)	0.16211 (15)	0.45487 (4)	0.0235 (3)	
O3	1.30215 (10)	−0.02588 (16)	0.74125 (4)	0.0257 (3)	
O4	1.43731 (10)	0.12427 (16)	0.70964 (4)	0.0254 (3)	
O5	0.93339 (9)	0.20943 (14)	0.63656 (4)	0.0213 (3)	
O6	0.48783 (9)	0.96225 (13)	0.55457 (4)	0.0191 (3)	
O7	0.59262 (10)	0.66818 (14)	0.45009 (4)	0.0231 (3)	
O8	0.78053 (10)	0.54250 (16)	0.74248 (4)	0.0240 (3)	
O9	0.91765 (10)	0.68595 (16)	0.71014 (5)	0.0259 (3)	
O10	0.43936 (9)	0.69818 (14)	0.63514 (4)	0.0200 (3)	
O11	0.47459 (11)	0.57361 (17)	0.72096 (5)	0.0309 (4)	
H11	0.463 (2)	0.608 (3)	0.6959 (5)	0.074 (11)*	
N1	1.04366 (11)	0.31279 (16)	0.50634 (5)	0.0165 (3)	
H1	1.0329 (17)	0.378 (2)	0.4864 (6)	0.038 (7)*	
N2	1.04156 (11)	0.25128 (16)	0.57939 (5)	0.0154 (3)	
N3	0.54195 (11)	0.81224 (16)	0.50323 (5)	0.0154 (3)	
H3	0.5312 (17)	0.8795 (19)	0.4838 (6)	0.034 (7)*	
N4	0.54112 (11)	0.74223 (16)	0.57559 (5)	0.0141 (3)	
C1	1.02324 (12)	0.35184 (19)	0.54753 (6)	0.0154 (4)	
C2	1.08106 (13)	0.1838 (2)	0.49329 (6)	0.0173 (4)	
C3	1.10081 (12)	0.08265 (19)	0.52809 (6)	0.0163 (4)	
C4	1.08091 (12)	0.11816 (19)	0.56934 (6)	0.0152 (4)	
C5	1.14720 (13)	−0.0542 (2)	0.51478 (7)	0.0195 (4)	
H5A	1.1235	−0.0805	0.4854	0.023*	
H5B	1.1286	−0.1305	0.5349	0.023*	
C6	1.25569 (14)	−0.0433 (2)	0.51483 (7)	0.0263 (5)	
H6A	1.2826	−0.1334	0.5051	0.039*	
H6B	1.2798	−0.0223	0.5442	0.039*	
H6C	1.2744	0.0328	0.4952	0.039*	
C7	1.10005 (13)	0.0217 (2)	0.60769 (6)	0.0168 (4)	
H7A	1.1054	−0.0771	0.5971	0.020*	
H7B	1.0449	0.0260	0.6270	0.020*	
C8	1.19039 (13)	0.05911 (19)	0.63389 (6)	0.0158 (4)	
C9	1.20206 (13)	0.0029 (2)	0.67599 (6)	0.0170 (4)	
H9	1.1522	−0.0518	0.6878	0.020*	
C10	1.28463 (13)	0.0257 (2)	0.70032 (6)	0.0172 (4)	
C11	1.35853 (13)	0.1076 (2)	0.68285 (6)	0.0175 (4)	
C12	1.34717 (13)	0.1648 (2)	0.64198 (6)	0.0178 (4)	
H12	1.3965	0.2210	0.6303	0.021*	
C13	1.26320 (13)	0.14051 (19)	0.61763 (6)	0.0171 (4)	
H13	1.2561	0.1805	0.5895	0.021*	
C14	1.23215 (15)	−0.1186 (2)	0.75862 (7)	0.0255 (5)	
H14A	1.2532	−0.1499	0.7876	0.038*	
H14B	1.2240	−0.2014	0.7397	0.038*	
H14C	1.1713	−0.0682	0.7605	0.038*	
C15	1.51657 (15)	0.1951 (3)	0.69177 (7)	0.0372 (6)	
H15A	1.5682	0.2020	0.7137	0.056*	
H15B	1.4974	0.2905	0.6825	0.056*	
H15C	1.5389	0.1416	0.6668	0.056*	
C16	1.01394 (14)	0.2859 (2)	0.62390 (6)	0.0188 (4)	
H16A	1.0680	0.2646	0.6442	0.023*	
H16B	1.0003	0.3887	0.6258	0.023*	
C17	0.84671 (14)	0.2553 (2)	0.61450 (7)	0.0217 (4)	
H17A	0.8526	0.2426	0.5828	0.026*	
H17B	0.8358	0.3572	0.6203	0.026*	
C18	0.76466 (15)	0.1707 (2)	0.62998 (7)	0.0222 (4)	
H18	0.7453	0.1883	0.6605	0.027*	
C19	0.75133 (16)	0.0224 (2)	0.61315 (7)	0.0266 (5)	
H19A	0.7254	−0.0494	0.6331	0.032*	
H19B	0.7987	−0.0143	0.5927	0.032*	
C20	0.68546 (15)	0.1388 (2)	0.59745 (7)	0.0263 (5)	
H20A	0.6193	0.1385	0.6078	0.032*	
H20B	0.6925	0.1736	0.5674	0.032*	
C21	0.52168 (13)	0.84630 (19)	0.54485 (6)	0.0152 (4)	
C22	0.57962 (13)	0.68532 (19)	0.48875 (6)	0.0158 (4)	
C23	0.60088 (12)	0.58061 (19)	0.52249 (6)	0.0150 (4)	
C24	0.58203 (12)	0.61172 (19)	0.56415 (6)	0.0141 (4)	
C25	0.64704 (13)	0.44552 (19)	0.50735 (6)	0.0179 (4)	
H25A	0.6224	0.4227	0.4778	0.021*	
H25B	0.6291	0.3668	0.5267	0.021*	
C26	0.75574 (14)	0.4565 (2)	0.50682 (7)	0.0260 (5)	
H26A	0.7823	0.3667	0.4967	0.039*	
H26B	0.7806	0.4767	0.5362	0.039*	
H26C	0.7739	0.5332	0.4873	0.039*	
C27	0.60529 (13)	0.51366 (19)	0.60141 (6)	0.0155 (4)	
H27A	0.6180	0.4177	0.5899	0.019*	
H27B	0.5492	0.5071	0.6201	0.019*	
C28	0.69145 (13)	0.56243 (19)	0.62879 (6)	0.0152 (4)	
C29	0.69588 (13)	0.52546 (19)	0.67285 (6)	0.0160 (4)	
H29	0.6460	0.4709	0.6848	0.019*	
C30	0.77194 (13)	0.5677 (2)	0.69894 (6)	0.0177 (4)	
C31	0.84680 (13)	0.6468 (2)	0.68105 (6)	0.0179 (4)	
C32	0.84309 (13)	0.6817 (2)	0.63778 (6)	0.0175 (4)	
H32	0.8937	0.7339	0.6256	0.021*	
C33	0.76497 (13)	0.64037 (19)	0.61165 (6)	0.0162 (4)	
H33	0.7623	0.6660	0.5819	0.019*	
C34	0.71250 (15)	0.4468 (2)	0.75976 (7)	0.0275 (5)	
H34A	0.7254	0.4345	0.7909	0.041*	
H34B	0.7172	0.3548	0.7452	0.041*	
H34C	0.6481	0.4852	0.7551	0.041*	
C35	0.98999 (16)	0.7776 (3)	0.69457 (8)	0.0404 (6)	
H35A	1.0358	0.7992	0.7182	0.061*	
H35B	0.9608	0.8658	0.6839	0.061*	
H35C	1.0230	0.7308	0.6709	0.061*	
C36	0.51807 (13)	0.7754 (2)	0.62070 (6)	0.0171 (4)	
H36A	0.5742	0.7541	0.6397	0.021*	
H36B	0.5044	0.8780	0.6231	0.021*	
C37	0.34872 (13)	0.7456 (2)	0.61695 (7)	0.0207 (4)	
H37A	0.3474	0.7335	0.5851	0.025*	
H37B	0.3392	0.8473	0.6235	0.025*	
C38	0.27141 (15)	0.6592 (2)	0.63636 (7)	0.0271 (5)	
H38	0.2615	0.6731	0.6680	0.033*	
C39	0.25401 (17)	0.5130 (2)	0.61916 (8)	0.0305 (5)	
H39A	0.2353	0.4388	0.6400	0.037*	
H39B	0.2945	0.4794	0.5955	0.037*	
C40	0.18343 (15)	0.6284 (2)	0.60890 (8)	0.0301 (5)	
H40A	0.1214	0.6254	0.6235	0.036*	
H40B	0.1807	0.6662	0.5789	0.036*	
C41	0.47636 (18)	0.6798 (3)	0.75372 (8)	0.0349 (6)	
H41A	0.4939	0.6350	0.7818	0.042*	
H41B	0.4114	0.7196	0.7561	0.042*	
C42	0.5443 (2)	0.7984 (3)	0.74559 (9)	0.0462 (7)	
H42A	0.5424	0.8666	0.7695	0.069*	
H42B	0.5261	0.8460	0.7184	0.069*	
H42C	0.6091	0.7604	0.7435	0.069*	

### Atomic displacement parameters (Å^2^)

**Table:**

	*U*^11^	*U*^22^	*U*^33^	*U*^12^	*U*^13^	*U*^23^
O1	0.0258 (7)	0.0163 (7)	0.0181 (7)	0.0056 (6)	0.0030 (6)	0.0036 (5)
O2	0.0315 (8)	0.0231 (7)	0.0162 (7)	0.0040 (6)	0.0058 (6)	0.0007 (6)
O3	0.0240 (7)	0.0370 (9)	0.0161 (8)	−0.0072 (7)	−0.0018 (6)	0.0090 (6)
O4	0.0208 (7)	0.0395 (9)	0.0158 (7)	−0.0102 (7)	−0.0029 (6)	0.0024 (6)
O5	0.0218 (7)	0.0232 (7)	0.0190 (7)	0.0047 (6)	0.0040 (6)	0.0066 (6)
O6	0.0240 (7)	0.0152 (7)	0.0182 (7)	0.0048 (6)	0.0023 (6)	0.0021 (5)
O7	0.0328 (8)	0.0225 (7)	0.0142 (7)	0.0042 (6)	0.0051 (6)	0.0021 (6)
O8	0.0258 (7)	0.0345 (8)	0.0117 (7)	−0.0098 (7)	−0.0018 (6)	0.0041 (6)
O9	0.0201 (7)	0.0390 (9)	0.0184 (8)	−0.0111 (7)	−0.0027 (6)	0.0011 (6)
O10	0.0223 (7)	0.0224 (7)	0.0153 (7)	0.0020 (6)	0.0017 (6)	0.0044 (6)
O11	0.0334 (9)	0.0315 (8)	0.0278 (9)	−0.0019 (7)	0.0007 (7)	0.0037 (7)
N1	0.0193 (8)	0.0158 (8)	0.0145 (8)	0.0024 (7)	0.0008 (6)	0.0045 (6)
N2	0.0164 (7)	0.0150 (8)	0.0148 (8)	0.0020 (6)	−0.0001 (6)	0.0030 (6)
N3	0.0194 (8)	0.0138 (8)	0.0130 (8)	0.0009 (7)	0.0007 (6)	0.0037 (6)
N4	0.0175 (7)	0.0130 (7)	0.0118 (8)	0.0013 (6)	0.0002 (6)	0.0011 (6)
C1	0.0126 (8)	0.0157 (9)	0.0179 (10)	−0.0012 (7)	−0.0001 (7)	0.0035 (7)
C2	0.0143 (9)	0.0170 (9)	0.0205 (10)	−0.0005 (8)	0.0011 (8)	0.0009 (8)
C3	0.0119 (8)	0.0148 (9)	0.0223 (10)	0.0007 (7)	0.0008 (7)	0.0033 (7)
C4	0.0117 (8)	0.0125 (8)	0.0213 (10)	−0.0017 (7)	−0.0020 (7)	0.0039 (7)
C5	0.0210 (9)	0.0177 (9)	0.0197 (10)	0.0015 (8)	0.0009 (8)	0.0014 (8)
C6	0.0207 (10)	0.0272 (11)	0.0308 (12)	0.0071 (9)	−0.0015 (9)	−0.0090 (9)
C7	0.0174 (9)	0.0156 (9)	0.0172 (10)	−0.0016 (8)	−0.0018 (8)	0.0046 (7)
C8	0.0164 (9)	0.0126 (8)	0.0184 (10)	0.0027 (7)	0.0009 (7)	−0.0004 (7)
C9	0.0163 (9)	0.0153 (9)	0.0195 (10)	0.0002 (8)	0.0031 (8)	0.0016 (7)
C10	0.0197 (9)	0.0204 (9)	0.0116 (9)	0.0016 (8)	0.0036 (7)	0.0009 (7)
C11	0.0171 (9)	0.0209 (9)	0.0145 (10)	−0.0007 (8)	0.0004 (7)	−0.0026 (8)
C12	0.0191 (9)	0.0166 (9)	0.0178 (10)	−0.0030 (8)	0.0037 (8)	−0.0002 (7)
C13	0.0210 (9)	0.0151 (9)	0.0153 (10)	0.0012 (8)	−0.0008 (8)	0.0016 (7)
C14	0.0265 (11)	0.0323 (11)	0.0178 (11)	−0.0043 (9)	0.0018 (8)	0.0073 (9)
C15	0.0244 (11)	0.0660 (17)	0.0210 (12)	−0.0180 (12)	−0.0012 (9)	0.0042 (11)
C16	0.0236 (10)	0.0167 (9)	0.0162 (10)	0.0007 (8)	0.0014 (8)	0.0023 (7)
C17	0.0228 (10)	0.0194 (9)	0.0229 (11)	0.0067 (8)	0.0048 (8)	0.0044 (8)
C18	0.0268 (10)	0.0213 (10)	0.0189 (11)	0.0027 (9)	0.0076 (8)	0.0001 (8)
C19	0.0357 (12)	0.0199 (10)	0.0244 (12)	0.0022 (9)	0.0040 (9)	0.0013 (9)
C20	0.0282 (11)	0.0248 (11)	0.0262 (12)	0.0044 (9)	0.0039 (9)	−0.0001 (9)
C21	0.0140 (8)	0.0157 (9)	0.0159 (10)	−0.0008 (7)	−0.0010 (7)	0.0022 (7)
C22	0.0141 (8)	0.0161 (9)	0.0172 (10)	−0.0014 (7)	0.0012 (7)	−0.0005 (7)
C23	0.0134 (9)	0.0134 (8)	0.0182 (10)	0.0001 (7)	−0.0008 (7)	0.0003 (7)
C24	0.0129 (8)	0.0127 (8)	0.0166 (10)	−0.0009 (7)	−0.0019 (7)	0.0010 (7)
C25	0.0199 (9)	0.0157 (9)	0.0181 (10)	0.0016 (8)	0.0003 (8)	−0.0015 (7)
C26	0.0200 (10)	0.0275 (11)	0.0306 (12)	0.0050 (9)	−0.0001 (9)	−0.0068 (9)
C27	0.0182 (9)	0.0142 (8)	0.0141 (10)	0.0010 (7)	−0.0020 (7)	0.0022 (7)
C28	0.0154 (9)	0.0128 (8)	0.0171 (10)	0.0026 (7)	−0.0017 (7)	−0.0001 (7)
C29	0.0180 (9)	0.0157 (9)	0.0146 (10)	0.0009 (8)	0.0016 (7)	0.0008 (7)
C30	0.0193 (9)	0.0209 (9)	0.0128 (9)	0.0010 (8)	0.0006 (7)	0.0014 (7)
C31	0.0164 (9)	0.0214 (9)	0.0158 (10)	−0.0015 (8)	−0.0008 (7)	−0.0024 (8)
C32	0.0162 (9)	0.0162 (9)	0.0201 (10)	−0.0019 (8)	0.0035 (8)	0.0005 (7)
C33	0.0205 (9)	0.0150 (9)	0.0130 (9)	0.0022 (8)	0.0011 (7)	0.0015 (7)
C34	0.0292 (11)	0.0380 (12)	0.0154 (11)	−0.0099 (10)	0.0021 (9)	0.0078 (9)
C35	0.0280 (12)	0.0652 (17)	0.0282 (13)	−0.0248 (12)	0.0025 (10)	−0.0014 (12)
C36	0.0212 (9)	0.0167 (9)	0.0135 (10)	0.0019 (8)	0.0000 (8)	0.0000 (7)
C37	0.0199 (9)	0.0206 (10)	0.0218 (11)	0.0043 (8)	0.0023 (8)	0.0008 (8)
C38	0.0323 (12)	0.0257 (11)	0.0238 (12)	0.0009 (9)	0.0084 (9)	−0.0031 (9)
C39	0.0375 (12)	0.0214 (10)	0.0330 (13)	0.0021 (10)	0.0073 (10)	−0.0011 (9)
C40	0.0248 (11)	0.0244 (11)	0.0412 (14)	0.0006 (9)	0.0059 (10)	−0.0044 (10)
C41	0.0371 (12)	0.0376 (13)	0.0304 (13)	−0.0013 (11)	0.0062 (10)	−0.0019 (10)
C42	0.0560 (17)	0.0367 (14)	0.0461 (17)	−0.0072 (13)	0.0057 (13)	−0.0022 (12)

### Geometric parameters (Å, °)

**Table:**

O1—C1	1.231 (2)	C16—H16B	0.9900
O2—C2	1.224 (2)	C17—C18	1.490 (3)
O3—C10	1.369 (2)	C17—H17A	0.9900
O3—C14	1.430 (2)	C17—H17B	0.9900
O4—C11	1.371 (2)	C18—C19	1.502 (3)
O4—C15	1.421 (3)	C18—C20	1.507 (3)
O5—C16	1.404 (2)	C18—H18	1.0000
O5—C17	1.443 (2)	C19—C20	1.506 (3)
O6—C21	1.232 (2)	C19—H19A	0.9900
O7—C22	1.223 (2)	C19—H19B	0.9900
O8—C30	1.366 (2)	C20—H20A	0.9900
O8—C34	1.427 (2)	C20—H20B	0.9900
O9—C31	1.371 (2)	C22—C23	1.460 (3)
O9—C35	1.426 (3)	C23—C24	1.352 (3)
O10—C36	1.405 (2)	C23—C25	1.509 (3)
O10—C37	1.446 (2)	C24—C27	1.504 (2)
O11—C41	1.422 (3)	C25—C26	1.529 (3)
O11—H11	0.853 (10)	C25—H25A	0.9900
N1—C1	1.361 (2)	C25—H25B	0.9900
N1—C2	1.389 (2)	C26—H26A	0.9800
N1—H1	0.879 (10)	C26—H26B	0.9800
N2—C1	1.385 (2)	C26—H26C	0.9800
N2—C4	1.409 (2)	C27—C28	1.526 (2)
N2—C16	1.474 (2)	C27—H27A	0.9900
N3—C21	1.361 (2)	C27—H27B	0.9900
N3—C22	1.387 (2)	C28—C33	1.383 (3)
N3—H3	0.884 (10)	C28—C29	1.403 (3)
N4—C21	1.387 (2)	C29—C30	1.377 (3)
N4—C24	1.407 (2)	C29—H29	0.9500
N4—C36	1.472 (2)	C30—C31	1.413 (3)
C2—C3	1.457 (3)	C31—C32	1.375 (3)
C3—C4	1.353 (3)	C32—C33	1.398 (3)
C3—C5	1.507 (3)	C32—H32	0.9500
C4—C7	1.511 (2)	C33—H33	0.9500
C5—C6	1.525 (3)	C34—H34A	0.9800
C5—H5A	0.9900	C34—H34B	0.9800
C5—H5B	0.9900	C34—H34C	0.9800
C6—H6A	0.9800	C35—H35A	0.9800
C6—H6B	0.9800	C35—H35B	0.9800
C6—H6C	0.9800	C35—H35C	0.9800
C7—C8	1.525 (2)	C36—H36A	0.9900
C7—H7A	0.9900	C36—H36B	0.9900
C7—H7B	0.9900	C37—C38	1.494 (3)
C8—C13	1.383 (3)	C37—H37A	0.9900
C8—C9	1.409 (3)	C37—H37B	0.9900
C9—C10	1.380 (3)	C38—C39	1.495 (3)
C9—H9	0.9500	C38—C40	1.506 (3)
C10—C11	1.411 (3)	C38—H38	1.0000
C11—C12	1.377 (3)	C39—C40	1.499 (3)
C12—C13	1.398 (3)	C39—H39A	0.9900
C12—H12	0.9500	C39—H39B	0.9900
C13—H13	0.9500	C40—H40A	0.9900
C14—H14A	0.9800	C40—H40B	0.9900
C14—H14B	0.9800	C41—C42	1.495 (4)
C14—H14C	0.9800	C41—H41A	0.9900
C15—H15A	0.9800	C41—H41B	0.9900
C15—H15B	0.9800	C42—H42A	0.9800
C15—H15C	0.9800	C42—H42B	0.9800
C16—H16A	0.9900	C42—H42C	0.9800
			
C10—O3—C14	117.09 (15)	C19—C20—H20B	117.8
C11—O4—C15	116.48 (16)	C18—C20—H20B	117.8
C16—O5—C17	112.87 (14)	H20A—C20—H20B	114.9
C30—O8—C34	115.82 (15)	O6—C21—N3	122.06 (17)
C31—O9—C35	116.85 (16)	O6—C21—N4	122.08 (17)
C36—O10—C37	114.01 (14)	N3—C21—N4	115.86 (16)
C41—O11—H11	112 (2)	O7—C22—N3	119.79 (17)
C1—N1—C2	126.77 (16)	O7—C22—C23	125.01 (17)
C1—N1—H1	115.4 (17)	N3—C22—C23	115.20 (17)
C2—N1—H1	117.8 (17)	C24—C23—C22	119.35 (17)
C1—N2—C4	121.31 (16)	C24—C23—C25	125.07 (17)
C1—N2—C16	117.50 (15)	C22—C23—C25	115.53 (17)
C4—N2—C16	121.12 (15)	C23—C24—N4	121.35 (16)
C21—N3—C22	126.78 (16)	C23—C24—C27	123.35 (17)
C21—N3—H3	115.9 (16)	N4—C24—C27	115.28 (16)
C22—N3—H3	117.3 (16)	C23—C25—C26	112.49 (16)
C21—N4—C24	121.41 (16)	C23—C25—H25A	109.1
C21—N4—C36	116.93 (15)	C26—C25—H25A	109.1
C24—N4—C36	121.66 (15)	C23—C25—H25B	109.1
O1—C1—N1	121.72 (17)	C26—C25—H25B	109.1
O1—C1—N2	122.30 (18)	H25A—C25—H25B	107.8
N1—C1—N2	115.98 (16)	C25—C26—H26A	109.5
O2—C2—N1	119.93 (17)	C25—C26—H26B	109.5
O2—C2—C3	124.93 (18)	H26A—C26—H26B	109.5
N1—C2—C3	115.15 (17)	C25—C26—H26C	109.5
C4—C3—C2	119.46 (17)	H26A—C26—H26C	109.5
C4—C3—C5	124.76 (17)	H26B—C26—H26C	109.5
C2—C3—C5	115.73 (17)	C24—C27—C28	112.94 (15)
C3—C4—N2	121.32 (17)	C24—C27—H27A	109.0
C3—C4—C7	123.46 (17)	C28—C27—H27A	109.0
N2—C4—C7	115.21 (16)	C24—C27—H27B	109.0
C3—C5—C6	112.34 (16)	C28—C27—H27B	109.0
C3—C5—H5A	109.1	H27A—C27—H27B	107.8
C6—C5—H5A	109.1	C33—C28—C29	119.28 (17)
C3—C5—H5B	109.1	C33—C28—C27	122.37 (17)
C6—C5—H5B	109.1	C29—C28—C27	118.35 (17)
H5A—C5—H5B	107.9	C30—C29—C28	120.49 (18)
C5—C6—H6A	109.5	C30—C29—H29	119.8
C5—C6—H6B	109.5	C28—C29—H29	119.8
H6A—C6—H6B	109.5	O8—C30—C29	124.77 (18)
C5—C6—H6C	109.5	O8—C30—C31	115.46 (16)
H6A—C6—H6C	109.5	C29—C30—C31	119.74 (17)
H6B—C6—H6C	109.5	O9—C31—C32	125.38 (18)
C4—C7—C8	113.77 (15)	O9—C31—C30	114.77 (17)
C4—C7—H7A	108.8	C32—C31—C30	119.82 (17)
C8—C7—H7A	108.8	C31—C32—C33	120.12 (18)
C4—C7—H7B	108.8	C31—C32—H32	119.9
C8—C7—H7B	108.8	C33—C32—H32	119.9
H7A—C7—H7B	107.7	C28—C33—C32	120.53 (18)
C13—C8—C9	118.39 (17)	C28—C33—H33	119.7
C13—C8—C7	123.16 (17)	C32—C33—H33	119.7
C9—C8—C7	118.39 (16)	O8—C34—H34A	109.5
C10—C9—C8	121.17 (18)	O8—C34—H34B	109.5
C10—C9—H9	119.4	H34A—C34—H34B	109.5
C8—C9—H9	119.4	O8—C34—H34C	109.5
O3—C10—C9	124.96 (17)	H34A—C34—H34C	109.5
O3—C10—C11	115.58 (16)	H34B—C34—H34C	109.5
C9—C10—C11	119.46 (17)	O9—C35—H35A	109.5
O4—C11—C12	125.36 (17)	O9—C35—H35B	109.5
O4—C11—C10	114.87 (17)	H35A—C35—H35B	109.5
C12—C11—C10	119.77 (17)	O9—C35—H35C	109.5
C11—C12—C13	120.17 (18)	H35A—C35—H35C	109.5
C11—C12—H12	119.9	H35B—C35—H35C	109.5
C13—C12—H12	119.9	O10—C36—N4	112.66 (15)
C8—C13—C12	121.02 (18)	O10—C36—H36A	109.1
C8—C13—H13	119.5	N4—C36—H36A	109.1
C12—C13—H13	119.5	O10—C36—H36B	109.1
O3—C14—H14A	109.5	N4—C36—H36B	109.1
O3—C14—H14B	109.5	H36A—C36—H36B	107.8
H14A—C14—H14B	109.5	O10—C37—C38	108.41 (16)
O3—C14—H14C	109.5	O10—C37—H37A	110.0
H14A—C14—H14C	109.5	C38—C37—H37A	110.0
H14B—C14—H14C	109.5	O10—C37—H37B	110.0
O4—C15—H15A	109.5	C38—C37—H37B	110.0
O4—C15—H15B	109.5	H37A—C37—H37B	108.4
H15A—C15—H15B	109.5	C39—C38—C37	118.25 (19)
O4—C15—H15C	109.5	C39—C38—C40	59.95 (14)
H15A—C15—H15C	109.5	C37—C38—C40	118.15 (19)
H15B—C15—H15C	109.5	C39—C38—H38	116.2
O5—C16—N2	112.32 (15)	C37—C38—H38	116.2
O5—C16—H16A	109.1	C40—C38—H38	116.2
N2—C16—H16A	109.1	C38—C39—C40	60.39 (14)
O5—C16—H16B	109.1	C38—C39—H39A	117.7
N2—C16—H16B	109.1	C40—C39—H39A	117.7
H16A—C16—H16B	107.9	C38—C39—H39B	117.7
O5—C17—C18	109.67 (16)	C40—C39—H39B	117.7
O5—C17—H17A	109.7	H39A—C39—H39B	114.9
C18—C17—H17A	109.7	C39—C40—C38	59.66 (14)
O5—C17—H17B	109.7	C39—C40—H40A	117.8
C18—C17—H17B	109.7	C38—C40—H40A	117.8
H17A—C17—H17B	108.2	C39—C40—H40B	117.8
C17—C18—C19	118.52 (18)	C38—C40—H40B	117.8
C17—C18—C20	117.06 (18)	H40A—C40—H40B	114.9
C19—C18—C20	60.09 (14)	O11—C41—C42	113.9 (2)
C17—C18—H18	116.4	O11—C41—H41A	108.8
C19—C18—H18	116.4	C42—C41—H41A	108.8
C20—C18—H18	116.4	O11—C41—H41B	108.8
C18—C19—C20	60.12 (13)	C42—C41—H41B	108.8
C18—C19—H19A	117.8	H41A—C41—H41B	107.7
C20—C19—H19A	117.8	C41—C42—H42A	109.5
C18—C19—H19B	117.8	C41—C42—H42B	109.5
C20—C19—H19B	117.8	H42A—C42—H42B	109.5
H19A—C19—H19B	114.9	C41—C42—H42C	109.5
C19—C20—C18	59.79 (13)	H42A—C42—H42C	109.5
C19—C20—H20A	117.8	H42B—C42—H42C	109.5
C18—C20—H20A	117.8		
			
C2—N1—C1—O1	−179.83 (17)	C22—N3—C21—O6	−179.73 (17)
C2—N1—C1—N2	−0.3 (3)	C22—N3—C21—N4	0.0 (3)
C4—N2—C1—O1	−179.19 (16)	C24—N4—C21—O6	−178.35 (16)
C16—N2—C1—O1	3.8 (3)	C36—N4—C21—O6	0.9 (3)
C4—N2—C1—N1	1.3 (2)	C24—N4—C21—N3	1.9 (2)
C16—N2—C1—N1	−175.71 (15)	C36—N4—C21—N3	−178.83 (15)
C1—N1—C2—O2	179.22 (17)	C21—N3—C22—O7	178.62 (17)
C1—N1—C2—C3	−0.8 (3)	C21—N3—C22—C23	−1.4 (3)
O2—C2—C3—C4	−179.02 (18)	O7—C22—C23—C24	−179.12 (18)
N1—C2—C3—C4	1.0 (2)	N3—C22—C23—C24	0.9 (2)
O2—C2—C3—C5	3.7 (3)	O7—C22—C23—C25	3.3 (3)
N1—C2—C3—C5	−176.27 (15)	N3—C22—C23—C25	−176.67 (15)
C2—C3—C4—N2	−0.1 (3)	C22—C23—C24—N4	0.9 (3)
C5—C3—C4—N2	176.91 (16)	C25—C23—C24—N4	178.20 (16)
C2—C3—C4—C7	−179.10 (16)	C22—C23—C24—C27	−177.24 (16)
C5—C3—C4—C7	−2.1 (3)	C25—C23—C24—C27	0.1 (3)
C1—N2—C4—C3	−1.1 (3)	C21—N4—C24—C23	−2.4 (3)
C16—N2—C4—C3	175.80 (16)	C36—N4—C24—C23	178.37 (16)
C1—N2—C4—C7	177.94 (15)	C21—N4—C24—C27	175.87 (15)
C16—N2—C4—C7	−5.1 (2)	C36—N4—C24—C27	−3.4 (2)
C4—C3—C5—C6	−89.8 (2)	C24—C23—C25—C26	−89.8 (2)
C2—C3—C5—C6	87.3 (2)	C22—C23—C25—C26	87.6 (2)
C3—C4—C7—C8	100.9 (2)	C23—C24—C27—C28	105.5 (2)
N2—C4—C7—C8	−78.2 (2)	N4—C24—C27—C28	−72.8 (2)
C4—C7—C8—C13	−19.6 (3)	C24—C27—C28—C33	−29.1 (2)
C4—C7—C8—C9	163.29 (17)	C24—C27—C28—C29	151.34 (17)
C13—C8—C9—C10	−1.2 (3)	C33—C28—C29—C30	0.8 (3)
C7—C8—C9—C10	176.06 (17)	C27—C28—C29—C30	−179.64 (17)
C14—O3—C10—C9	4.7 (3)	C34—O8—C30—C29	10.2 (3)
C14—O3—C10—C11	−175.13 (17)	C34—O8—C30—C31	−171.77 (17)
C8—C9—C10—O3	−179.42 (18)	C28—C29—C30—O8	176.90 (17)
C8—C9—C10—C11	0.4 (3)	C28—C29—C30—C31	−1.0 (3)
C15—O4—C11—C12	−6.3 (3)	C35—O9—C31—C32	4.1 (3)
C15—O4—C11—C10	174.49 (19)	C35—O9—C31—C30	−174.06 (19)
O3—C10—C11—O4	−0.3 (3)	O8—C30—C31—O9	0.4 (3)
C9—C10—C11—O4	179.84 (17)	C29—C30—C31—O9	178.56 (17)
O3—C10—C11—C12	−179.57 (17)	O8—C30—C31—C32	−177.86 (17)
C9—C10—C11—C12	0.6 (3)	C29—C30—C31—C32	0.3 (3)
O4—C11—C12—C13	−179.94 (18)	O9—C31—C32—C33	−177.37 (18)
C10—C11—C12—C13	−0.8 (3)	C30—C31—C32—C33	0.7 (3)
C9—C8—C13—C12	1.0 (3)	C29—C28—C33—C32	0.2 (3)
C7—C8—C13—C12	−176.10 (17)	C27—C28—C33—C32	−179.34 (17)
C11—C12—C13—C8	−0.1 (3)	C31—C32—C33—C28	−1.0 (3)
C17—O5—C16—N2	−70.73 (19)	C37—O10—C36—N4	−73.12 (19)
C1—N2—C16—O5	106.91 (18)	C21—N4—C36—O10	108.98 (17)
C4—N2—C16—O5	−70.1 (2)	C24—N4—C36—O10	−71.7 (2)
C16—O5—C17—C18	−179.34 (16)	C36—O10—C37—C38	−177.59 (16)
O5—C17—C18—C19	−77.3 (2)	O10—C37—C38—C39	−78.7 (2)
O5—C17—C18—C20	−146.26 (17)	O10—C37—C38—C40	−147.80 (18)
C17—C18—C19—C20	−106.5 (2)	C37—C38—C39—C40	−107.9 (2)
C17—C18—C20—C19	109.0 (2)	C37—C38—C40—C39	108.1 (2)

### Hydrogen-bond geometry (Å, °)

**Table:**

*D*—H···*A*	*D*—H	H···*A*	*D*···*A*	*D*—H···*A*
N1—H1···O1^i^	0.88 (1)	1.96 (1)	2.839 (2)	177 (2)
N3—H3···O6^ii^	0.88 (1)	1.92 (1)	2.799 (2)	175 (2)
O11—H11···O10	0.85 (1)	2.08 (1)	2.927 (2)	178 (1)

Symmetry codes: (i) −*x*+2, −*y*+1, −*z*+1; (ii) −*x*+1, −*y*+2, −*z*+1.
